# Quantum mechanical polar surface area

**DOI:** 10.1007/s10822-012-9557-y

**Published:** 2012-03-04

**Authors:** Gijs Schaftenaar, Jakob de Vlieg

**Affiliations:** CMBI, Computational Drug Discovery Group, Nijmegen Centre for Life Sciences, Nijmegen University, Geert-Grooteplein 28, 6525 Nijmegen, GA The Netherlands

**Keywords:** Drug discovery, Computational chemistry, Polar Surface Area

## Abstract

A correlation has been established between the absorbed fraction of training-set molecules after oral administration in humans and the Quantum Mechanical Polar Surface Area (QMPSA). This correlation holds for the QMPSA calculated with structures where carboxyl groups are deprotonated. The correlation of the absorbed fraction and the QMPSA calculated on the neutral gas phase optimized structures is much less pronounced. This suggests that the absorption process is mainly determined by polar interactions of the drug molecules in water solution. Rules are given to derive the optimal polar/apolar ranges of the electrostatic potential.

## Introduction

The polar surface area (PSA) has been used successfully to predict the absorption of drugs [1]. The polar surface area is defined as the combined surface area belonging to oxygen and nitrogen atoms and hydrogen atoms bound to these electronegative atoms. Methods to improve the correlation between polar surface area and absorption of drugs evolved in the years thereafter. The Dynamic Polar Surface Area (DPSA) is derived from Boltzmann-averaged ensembles of low energy molecular conformations [2]. The Topological Polar Surface Area (TPSA) is fragment-based methodology which derived standardized contributions to the molecular polar surface area from functional groups and atom types [3]. Various protocols have been reported to calculate the PSA on different surfaces (van der Waals [1], Connolly, or solvent accessible surface [4].

The term polar surface area suggests that the absorption is related to the physical interaction of surfaces through their electrostatic potential. In this work we present a study of the correlation of the quantum mechanical electrostatic potential and the absorption of drugs in humans. In the original work by Palm et al. [1] the surface was constructed by intersecting atomic spheres defined by van der Waals radii. In line with our quantum mechanical approach in this work the electrostatic potential will be calculated on a surface with constant electron density or isodensity surface.

The algorithm by Palm et al. was incorporated to our MOLDEN molecular modeling package [5] for comparison. Choices have to be made about which value of the electron density the isodensity surface is most suited to calculate the electrostatic potential.

Secondly, a range of the electrostatic potential has to be defined as polar and a complementary range as apolar. The sum of all parts of the isodensity surface with an electrostatic potential in the polar range is then defined as the polar surface area.

## Methods

The structures of the training-set molecules were kindly provided by Popelier et al. [6], the authors of a paper on the quantum chemical calculation of the topological polar surface area. For a detailed description on how these structures were derived, we refer to this paper [6].

Below a concise summary on how these structures were calculated and used in this work. Low-energy conformations for each molecule in the training-set were obtained from Monte Carlo multiple minimum (MCMM) searching, using the OPLS-AA force field.

The MCMM [7]/OPLS-AA [8] geometries were used as the starting point for the quantum mechanical geometry optimizations at the B3LYP/6–31G**//B3LYP/6–31G** level of theory [9] using the Jaguar program [10].

A validation set of compounds with absorbance data in humans, was obtained from J.Kelder et al. [11]. The validation set will be used to determine whether the optimized ranges of the electrostatic potential for polar and apolar surface are also valid for an independent set of molecules.

Single-point energy calculations were performed with the optimized geometries by the program Gamess-US [12] at the B3LYP/6–31G** level, to generate the wave function files required for the calculations of the electron density and electrostatic potential on a three dimensional grid or cube file with the Molden (version 4.7) program [5].

These cube files are subsequently used to map the electrostatic potential onto an isodensity surface with the Molden program. The isodensity surface is represented as a collection of triangles, calculated with the marching cube algorithm implemented in Molden. The electrostatic potential of the vertices of each triangle is interpolated from the potential of the eight grid points of the cube marching [13] over the three dimensional grid. The polar surface area is calculated as the sum of the triangular areas with the potential in the polar range. When not all vertices are in the polar range the triangles are subdivided into four smaller triangles. This process is repeated until all vertices are either in the polar range or all in the apolar range.

Sigmoidal fits between the QMPSA and the fraction absorbed in humans (FA) were performed using the four parameter Weibull equation:

$$ {\text{FA}} = {\text{a}}-{\text{b}}{*} \exp \left( { - {\text{c}}{*} {\text{QMPSA}}^{\text{d}} } \right) $$

Where a, b, c and d are parameters to be fitted.

## Results and discussion

### Isodensity surfaces

Varying the value of the electron density of the isodensity we established that a value of 0.0005 electrons/bohr^3^ would give a surface most compatible with the van der waals surface used by Palm et al. [1]. Table 1 shows the total surface area for the training-set molecules with both methods. The root mean square deviation is 9 Å^2^ which is around 3%.

**Table 1:** Comparison between the quantum mechanical total surface area of the isodensity surface at 0.0005 electrons/bohr3 and the topological surface area by Palm et al. [1]

Drug	Quantum mechanical surface area (Å^2^)	Topological surface area (Å^2^)
Metoprolol	382	385
Nordiazepam	312	311
Diazepam	329	332
Oxprenolol	354	372
Phenazone	253	248
Oxazepam	320	317
Alprenolol	354	360
Practolol	369	365
Pindolol	342	335
Ciprofloxacin	364	364
Metolazone	383	385
Tranexamic	218	213
Atenolol	367	364
Sulpiride	407	407
Mannitol	224	216
Foscarnet	143	130
Sulfasalazine	432	415
Olsalazine	336	315

The electron density that best matches the van der Waals surface is relatively low. Quantum mechanical methods optimize the electron density with respect to the energy.

An artifact of these methods is that the contribution to the electron density by energy rich inner shell electrons/orbitals is optimized at the expense of that of the outer shell and valence electrons/orbitals, when not using a complete basis-set [14]. In the same spirit it can be argued that the electron density at locations that contribute higher to the energy is optimized at the expense of quality of the electron density at locations that contribute less to the energy. In order to avoid/evaluate this complication the Quantum Mechanical Polar Surface Area (QMPSA) will also be evaluated on isodensity surfaces with a higher electron density.

### Polar and apolar range of electrostatic potential

**Fig. 1:**
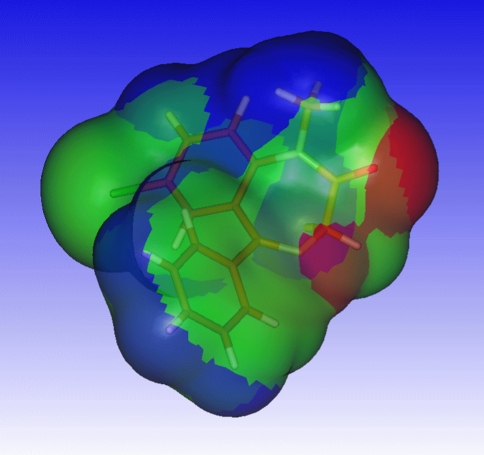
Quantum mechanical polar surface area (*red* and *blue*) for the training-set molecule diazepam when choosing the apolar electrostatic potential range incorrectly (ESP_apolar,high_ 0.01, ESP_apolar,low_ −0.028)

**Fig. 2:**
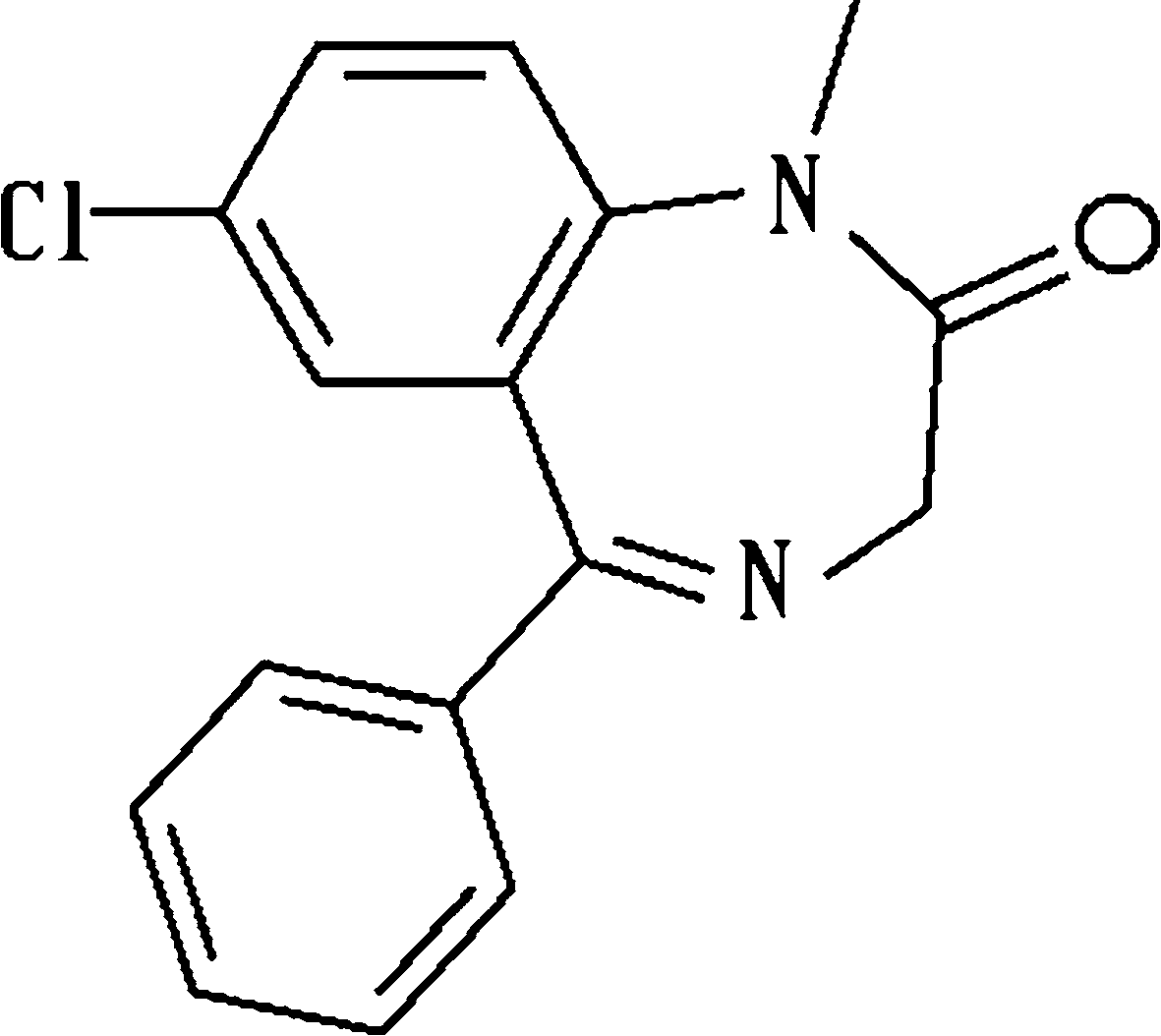
Structural formula diazepam

Experimenting with upper and lower bound of the electrostatic potential defined as apolar, the following observations were made. Choosing the upper bound of the apolar electrostatic potential (ESP_apolar,high_) too low results in hydrogens connected to phenyl rings to contribute to polar surface with positive electrostatic potential (see Fig. 1 and Fig. 2). Conversely, choosing the lower bound of the apolar potential (ESP_apolar,low_) too high, results in the electron density above and under the phenyl rings to contribute to the polar surface area with negative electrostatic potential. Phenyl rings constitute a major part of the isodensity surface, and since these atoms do not contribute to the topological polar surface area, ESP_apolar,low_ and ESP_apolar,high_ were chosen such that the phenyl rings do not contribute to the Quantum Mechanical Polar Surface Area (QMPSA).

### Correlation between QMPSA and the absorbed fraction of training-set molecules

Table 2 shows the calculated QMPSA and the absorbed fraction (FA) of traning-set molecules [1], on the isodensity surfaces with density values 0.01 and 0.0005 e/Bohr^3^. Upper and lower bound of the polar electrostatic potential were chosen according to the rules set out in the previous section and were optimized to yield the highest correlation coefficient between the QMPSA and the absorbed fraction of the training-set molecules.

**Table 2:** QMPSA calculated at different density values versus absorbed fraction of the gasphase optimized training-set molecules

Drug	QMPSA (Å^2^)	QMPSA (Å^2^)	Absorbed Fraction
	Density 0.01 e/bohr^3^ ESP_apolar,low_ −0.028 ESP_apolar,high_ 0.115	Density 0.0005 e/bohr^3^ ESP_apolar,low_ −0.025 ESP_apolar,high_ 0.043	
Metoprolol	20.8	31.1	102
Nordiazepam	18.4	35.0	99
Diazepam	15.2	29.6	97
Oxprenolol	19.5	27.1	97
Phenazone	13.0	33.7	97
Oxazepam	26.2	48.0	97
Alprenolol	15.3	14.3	96
Practolol	25.7	37.6	95
Pindolol	17.6	43.4	92
Ciprofloxacin	34.3	57.5	69
Metolazone	46.6	97.2	64
Tranexamic	20.3	28.9	55
Atenolol	28.2	39.7	54
Sulpiride	45.3	80.8	36
Mannitol	40.2	48.6	26
Foscarnet	35.1	42.4	17
Sulfasalazine	37.7	49.8	12
Olsalazine	27.7	30.2	2.3

The correlation between the absorbed fraction of the training-set molecules and the QMPSA is relatively low compared to the reported sigmoidal correlation between topological polar surface area and the absorbed fraction. Correlation coefficients for a linear fit are 0.68 and 0.30 for density values 0.01 and 0.0005 e/bohr^3^ respectively. Correlation coefficients for a sigmoidal fit are slightly better: 0.74 and 0.36 for density values 0.01 and 0.0005 e/bohr^3^ respectively.

**Fig. 3:**
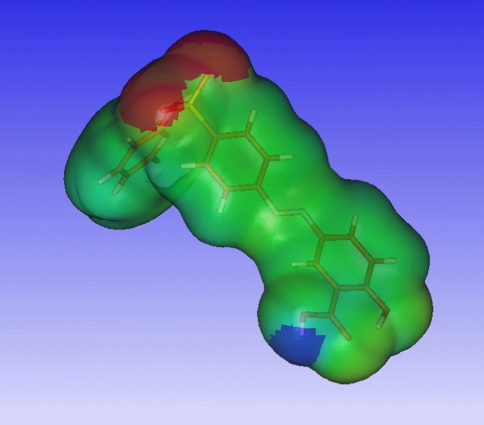
Quantum mechanical polar surface area (*red and blue*) for the training-set molecule sulfasalazine

**Fig. 4:**
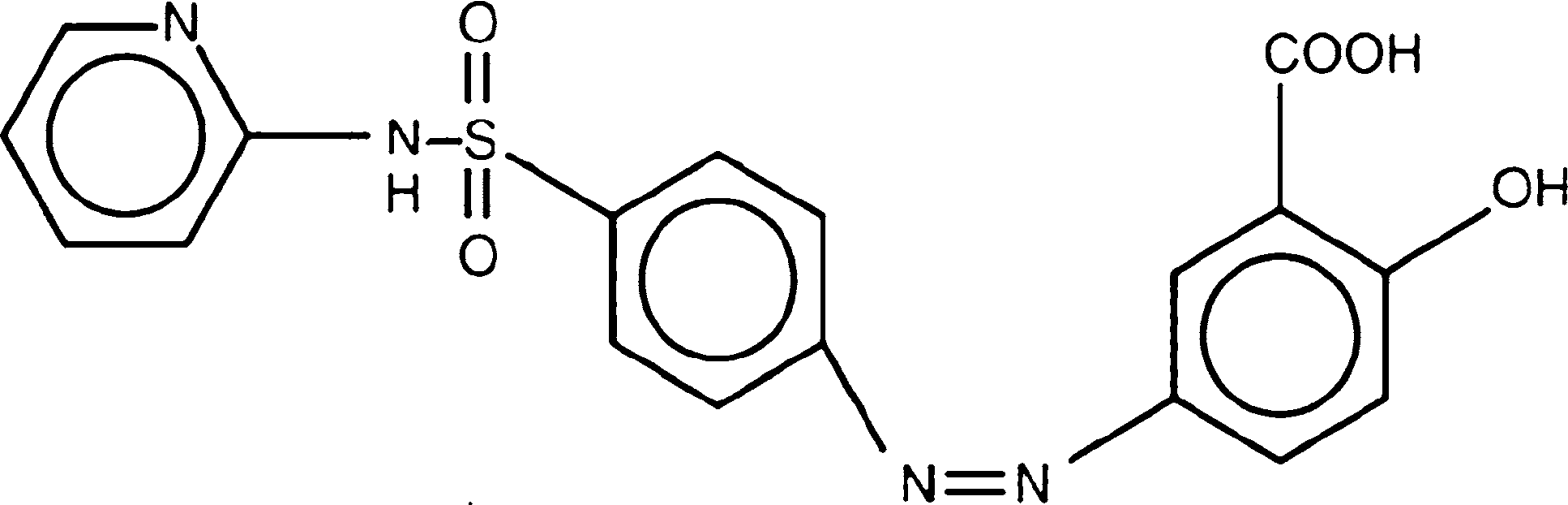
Structural formula sulfasalazine

A graphical inspection of the QMPSA revealed the reason for the often relatively low values of the QMPSA with respect to the topological polar surface area. Figure 3 shows the electrostatic potential mapped onto the isodensity surface for the training-set molecule sulfasalazine. Figure 4 shows the structural formula of sulfasalazine. QMPSA surface areas are marked with the red and blue colors. Red and blue represent polar surface areas with respectively positive and negative electrostatic potentials. The hydrogen of hydroxyl group attached to the phenyl ring points towards the oxygen of the carboxyl group in the optimized sulfasalazine structure. The positive electrostatic potential exerted by the hydroxyl hydrogen cancels out the negative electrostatic potential exerted by the carboxyl oxygen. The resultant potential has a low absolute value and is therefore classified as an apolar potential.

In general optimized structures in the gas phase will tend to have their electronegative atoms (O, N) oriented towards electropositive counterparts (H), whereas molecules in a polar solvent such as water will tend to have both their electronegative and electropositive atoms accessible for interaction with the solvent.

The absorbed fraction pertains to the fraction of molecules in solution, absorbed into the apolar membranes of the gut. Gas phase optimized structures are therefore best suited to represent the absorbed state of the training-set molecules. The water solved state of the molecules can probably best be represented by taking into account the neutral species and the deprotonated species, with their electronegative atoms accessible to the solvent.

### Influence of the protonation state of acids

The carboxyl group of the molecules in our training-set can lose their proton depending on the pH with respect to the acid’s p*K*_a_. In our training-set three molecules contain a carboxyl group: ciprofloxacin, sulfasalazine and olsalazine. The latter two have p*K*_a_’s such that the they are dissociated at the pH of the gut (pH = 5.7–6.6 [15]). Ciprofloxacin however has a p*K*_a_ that falls in the pH range of the gut (p*K*_a_ = 6.09 [16]). We assume therefore that half of the ciprofloxacin molecules are dissociated and the other half are not.

The QMPSA for these three molecules should (also) be calculated on the deprotonated species. The QMPSA of ions is dominated by the charge center and is therefore much larger than their neutral counterparts. For sulfasalazine for example the QMPSA for the anion is 198.7 Å^2^ versus 56.0 Å^2^ for the neutral species. In aqueous solution however, counter ions are always present at some distance. We optimized the anion-lithium complex to obtain an approximate distance for the counter ion to the central carbon of the carboxyl group (2.281 Å). Placing a positive point charge at this distance, the QMPSA for the anion-point charge complex was calculated. The QMPSA calculated in this way should be considered to be a lower bound for the QMPSA of the dissociated acid, since the distance of the counter charge will be larger in aqueous solution. The QMPSA for ciprofloxacin, sulfasalazine and olsalazine are 48.6, 59.8 and 62.8 Å^2^ respectively versus 34.4, 37.7 and 27.7 Å^2^ respectively in the neutral species. For ciprofloxacin we take the average of the anion and the neutral species: 41.8 Å^2^.

**Fig. 5:**
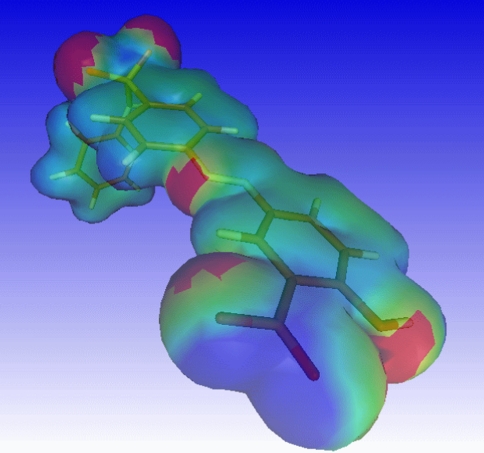
Quantum mechanical polar surface area (*red and blue*) for the test-set molecule sulfasalazine with carboxyl group deprotonated

Figure 5 shows the electrostatic potential mapped onto the isodensity surface for the deprotonated training-set molecule sulfasalazine.

Table 3 shows the calculated QMPSA and the absorbed fraction (FA) of training-set molecules with carboxyl groups deprotonated, on the isodensity surfaces with density values 0.01 and 0.0005 e/bohr^3^. The correlation between the absorbed fraction of the training-set molecules and the QMPSA is significantly better compared to that of the neutral gas phase optimized structures.

**Table 3:** QMPSA calculated at different density values versus absorbed fraction of the training-set molecules with deprotonated carboxyl groups

Drug	QMPSA (Å^2^)		Absorbed fraction
	Density 0.01 e/bohr^3^ ESP_apolar,low_ −0.028 ESP_apolar,high_ 0.115	Density 0.0005 e/bohr^3^ ESP_apolar,low_ −0.025 ESP_apolar,high_ 0.043	
Metoprolol	20.8	31.1	102
Nordiazepam	18.4	35.0	99
Diazepam	15.2	29.6	97
Oxprenolol	19.8	36.9	97
Phenazone	13.0	33.7	97
Oxazepam	26.2	48.0	97
Alprenolol	17.1	20.8	96
Practolol	29.5	45.5	95
Pindolol	21.3	43.4	92
Ciprofloxacin	41.8	83.8	69
Metolazone	46.6	97.2	64
Tranexamic	25.2	44.8	55
Atenolol	32.4	49.9	54
Sulpiride	45.3	80.8	36
Mannitol	57.1	78.5	26
Foscarnet	43.0	62.9	17
Sulfasalazine	59.8	118.3	12
Olsalazine	62.8	103.5	2.3

Correlation coefficients for a linear fit are 0.87 and 0.84 for density values 0.01 and 0.0005 e/bohr^3^ respectively. Correlation coefficients for a sigmoidal fit are also better: 0.92 and 0.86 for density values 0.01 and 0.0005 e/bohr^3^ respectively.

**Fig. 6:**
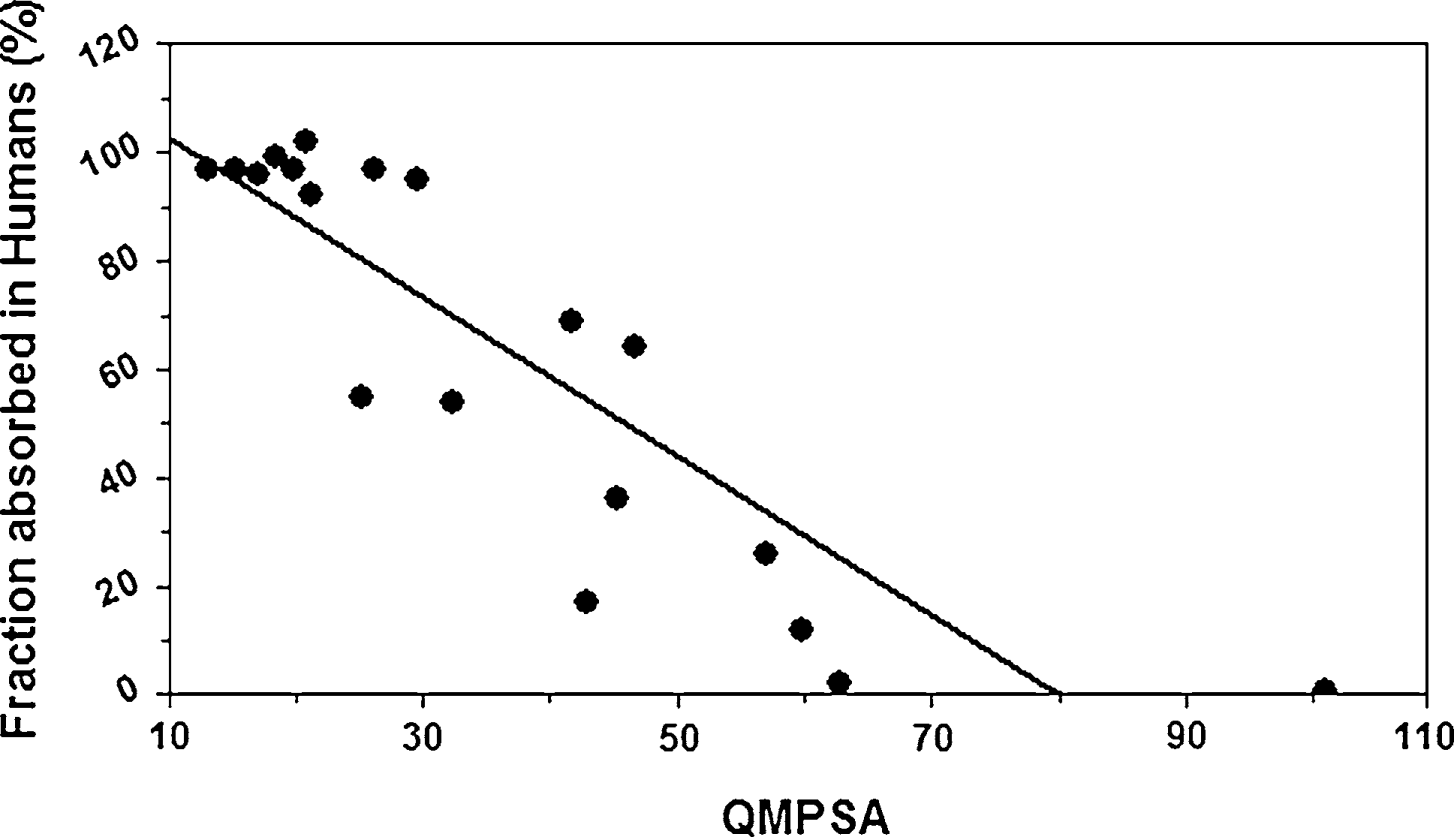
Linear correlation between the absorbed fraction of training-set molecules with deprotonated carboxyl groups and the QMPSA

**Fig. 7:**
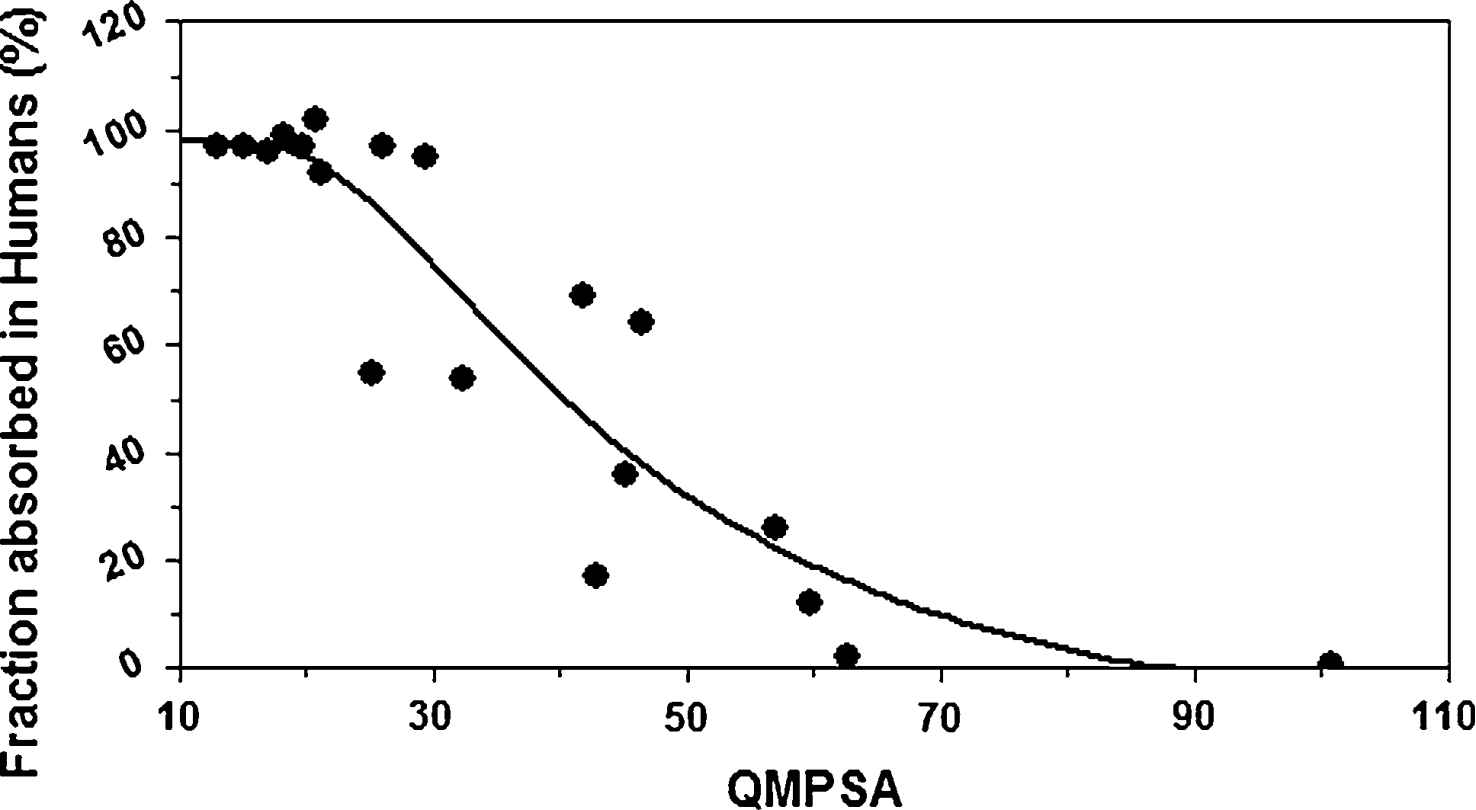
correlation between the absorbed fraction of training-set molecules with deprotonated carboxyl groups and the QMPSA

Figure 6 and Fig. 7 show respectively the linear and sigmoidal correlation between the absorbed fraction of training-set molecules with carboxyl groups deprotonated, and the QMPSA calculated on the isodensity surface with density value 0.01 e/bohr^3^.

### Correlation between QMPSA and the absorbed fraction of validation-set molecules

Table 4 shows the calculated QMPSA and the absorbed fraction (FA) of validation-set molecules with deprotonated carboxyl groups, on the isodensity surfaces with density values 0.01 and 0.0005 e/bohr^3^. Correlation coefficients for a linear fit are 0.96 and 0.95 for density values 0.01 and 0.0005 e/bohr^3^ respectively. Correlation coefficients for a sigmoidal fit are slightly better and worse: 0.98 and 0.87 for density values 0.01 and 0.0005 e/bohr^3^ respectively.

**Table 4:** QMPSA calculated at different density values versus absorbed fraction of the validation-set molecules

Drug	QMPSA (Å^2^)	QMPSA (Å^2^)	Absorbed fraction
	Density 0.01 e/bohr^3^ ESP_apolar, low_ −0.028 ESP_apolar, high_ 0.115	Density 0.0005 e/bohr^3^ ESP_apolar, low_ −0.025 ESP_apolar, high_ 0.043	
Caffeine	27.0	40.0	100
Salicylic acid	30.7	54.4	100
Norgestrel	22.1	37.9	100
Felodipine	30.5	38.0	100
Tiacrilast	30.9	42.3	99
Theophylline	32.2	51.9	98
Testosterone	21.9	40.5	98
Verapamil	33.3	58.3	95
Warfarine	28.2	54.7	93
Diltiazem	28.2	43.6	92
Propranolol	16.6	21.6	90
Hydrocortisone	42.0	62.1	89
Cimetidine	37.1	78.9	84
Terbutaline	32.7	53.4	73
Ceftriaxone	93.1	181.1	1
Aztreonam	98.8	155.9	0

**Fig. 8:**
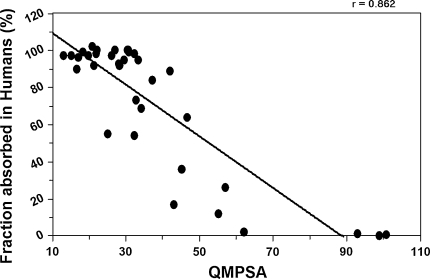
Linear correlation between the absorbed fraction of training + validation-set molecules and the QMPSA

**Fig. 9:**
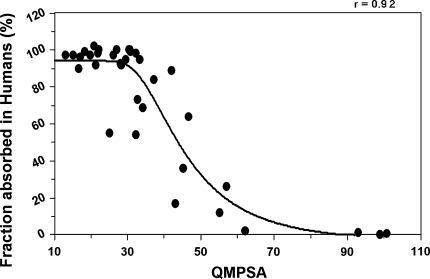
Sigmoidal correlation between the absorbed fraction of training + validation-set molecules and the QMPSA. Sigmoidal fit parameters: a = 94.05, b = 98.47, c = 3327398.1, d = −4.0328

Figure 8 and Fig. 9 show respectively the linear and sigmoidal correlation between the absorbed fraction of combined validation- and training-set molecules and the QMPSA calculated on the isodensity surface with density value 0.01 e/bohr^3^, with correlation coefficients of 0.86 and 0.92 respectively. The combined set shows an equally good sigmoidal correlation compared to the training-set alone (0.92 versus 0.92). Although the isodensity surfaces of 0.0005 e/bohr^3^ best represents the van der Waals surfaces used in the original work of Palm et al., for the calculation of the QMPSA the use of 0.01 e/bohr^3^ isodensity surfaces consistently give better fits and are therefore recommended together with ESP_apolar,low_ of −0.028 and ESP_apolar,high_ of 0.115.

### Relation between gas phase and solvent optimised structures

To assess the influence of gas phase versus solvent optimized structures on the correlation between QMPSA and absorbed fraction in humans, the training set of molecules was optimized with an explicit water for each hydrogen bond donor using the Polarizable Continuum solvent Model (PCM) [17]. The 4–31G* basis set was used at the Hartree–Fock level of theory. After optimization the explicit waters are removed and the QMPSA is calculated at the B3LYP/6–31G** level of theory at the PCM optimized geometries.

Table 5 shows the results. Comparing with the gas phase approach, the QMPSA changes are small (RMSD 1.73 Å^2^). Correlation coefficients for a linear and sigmoidal fit are 0.86 and 0.91 respectively for isodensity values 0.01 e/bohr^3^ (versus 0.87 and 0.92 respectively for gas phase approach).

**Table 5:** QMPSA calculated at density value 0.01 e/bohr^3^ versus absorbed fraction of the test-set molecules with deprotonated carboxyl groups optimized with the PCM model at the Hartree–Fock level of theory with the 4–31G* basis-set

Drug	QMPSA (Å^2^)	Absorbed fraction
	Density 0.01 e/bohr^3^ ESP_apolar,low_ −0.028 ESP_apolar,high_ 0.115	
Metoprolol	20.9	102
Nordiazepam	18.4	99
Diazepam	15.7	97
Oxprenolol	23.2	97
Phenazone	14.0	97
Oxazepam	27.3	97
Alprenolol	14.7	96
Practolol	28.4	95
Pindolol	20.7	92
Ciprofloxacin	48.6	69
Metolazone	47.3	64
Tranexamic	25.2	55
Atenolol	31.5	54
Sulpiride	46.0	36
Mannitol	55.7	26
Foscarnet	39.6	17
Sulfasalazine	59.8	12
Olsalazine	62.8	2.3

The correlation between QMPSA and absorbed fraction in humans is expected to improve when using ensembles of low energy molecular conformations as in the Dynamic Polar Surface Area method [2], but was not further investigated.

**Fig. 10:**
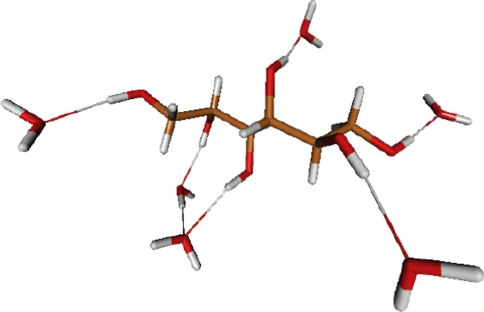
PCM/4–31G* optimized structure of mannitol with six explicit water molecules

Figure 10 mannitol with six explicit waters optimized with the PCM solvent model at the Hartree–Fock level of theory with the 4–31G* basis set.

### QMPSA basis set and level of theory dependency

Table 6 shows that the QMPSA is in general weakly dependent on the used basis set. The root mean square deviation for nordiazepam, tranexamic and sulfasalazine is 0.14, 0.69 and 3.96 respectively (1, 2 and 5%) over the employed basis sets. The compounds were chosen to represent the apolar, medium polar to polar spectrum. The RMSD increases with the polarity of the compounds.

**Table 6:** The QMPSA calculated with different basis sets at the Hartree–Fock level of theory with the PCM solvent model optimized geometries

	QMPSA (Å^2^) Density 0.01 e/bohr^3^ ESP_apolar,low_ −0.028 ESP_apolar,high_ 0.115		
Drug/basis set	3–21G	4–31G	6–31G**
Nordiazepam	20.7	20.7	21.0
Tranexamic	29.9	29.8	28.4
Sulfasalazine	73.4	73.2	64.9

Going from the Hartree–Fock level of theory to B3LYP, the QMPSA decreases by 10%(see Table 5 and Table 6 basis set 6–31G**). This is not surprising since the Hartree–Fock method is known to overestimate the polarity [18].

## Conclusions

A good correlation has been established between the absorbed fraction of training-set molecules after oral administration in humans and the Quantum Mechanical Polar Surface Area (QMPSA). This correlation holds for the QMPSA calculated with structures where the carboxyl groups are deprotonated. The correlation of the absorbed fraction and the QMPSA calculated on the gas phase optimized structures is much less pronounced. This suggests that the absorption process is mainly determined by polar interactions of the molecules in water solution.

The QMPSA is weakly dependent on the used basis set and drops 10% on going from Hartree–Fock to B3LYP.
